# Safety and Efficacy in HIV-1-Infected Patients Treated with Ritonavir-Boosted Saquinavir Mesylate

**DOI:** 10.1111/j.1753-5174.2009.00028.x

**Published:** 2010-03

**Authors:** Heribert Knechten, Thomas Lutz, Piotr Pulik, Teodoro Martin, Andre Tappe, Hans Jaeger

**Affiliations:** * Praxenzentrum BlondelstrasseAachen, Germany; † Infektiologikum Frankfurt, Frankfurt am MainGermany; ‡ Hospital for Infectious DiseasesWarsaw, Poland; § Hospital Puerta de HierroMadrid, Spain; ¶ Roche Pharma AG, Grenzach-WyhlenGermany; ** HIV Research and Clinical Care Center MunichMunich, Germany

**Keywords:** Bioavailability, Ritonavir, Safety, Saquinavir, Tolerability

## Abstract

**Objective:**

To evaluate the safety, tolerability, and efficacy of ritonavir-boosted saquinavir 1000/100 mg twice daily administered as a 500 mg film-coated tablet in HIV-1-infected patients.

**Methods:**

In this open-label, observational, 24-week survey conducted in 8 European countries, eligible HIV-infected participants had been prescribed saquinavir/ritonavir in combination with other nonprotease inhibitor (PI) antiretroviral agents as part of their HIV treatment regimen. The safety (grade 3 or 4 adverse events [AEs]), tolerability (by an investigator-reported subjective rating system), and efficacy (the percentage of participants with <50 and <400 copies/mL HIV RNA and change from baseline in mean CD4+ cell count) were analyzed for the overall study population and 7 subpopulations.

**Results:**

The enrolled population included 2122 participants with 1908 completing the study; 44 (2.1%) withdrew prematurely because of AEs, including 7 nontreatment-related deaths. There were 33 grade 3 or 4 AEs in 29 (1.4%) participants; 7 AEs in 7 (0.3%) participants were considered treatment-related. Tolerability was reported to be “very good” or “good” in 42% and 25% of participants, respectively. From baseline to week 24, the proportion of participants with HIV RNA <50 copies/mL increased from 31.2% to 47.6% and the proportion with <400 copies/mL increased from 42.5% to 61.4%; the mean CD4+ cell count increased by 75 cells/µL. In the subpopulation analysis, the greatest efficacy benefits occurred in participants who were treatment-naïve and in those not having received prior PI therapy.

**Conclusions:**

Treatment with the saquinavir 500 mg film-coated tablet resulted in few grade 3 or 4 AEs and was well tolerated and effective in a broad population of patients.

## Introduction

The current U.S. [1] and European [2] guidelines for the initial treatment of human immunodeficiency virus-1 (HIV-1)-infected patients recommend a regimen comprising 2 nucleoside reverse transcriptase inhibitors (NRTIs) and either a non-NRTI (NNRTI) or a protease inhibitor (PI) as components of highly active antiretroviral therapy (HAART). This recommendation is based on the considerable reduction in HIV-1-related morbidity and mortality since the introduction of HAART [3–5] and in particular the established efficacy of PIs [1,2].

Saquinavir mesylate (SQV; Invirase®, Roche, Inc) is a potent inhibitor of the HIV protease. SQV has been approved by the European Medicines Agency (EMEA) and the U.S. Food and Drug Administration (FDA) for the treatment of HIV infection in combination with other antiretroviral (ARV) agents. Available for oral administration as a 200 mg hard gelatin capsule and a 500 mg film-coated tablet [6], the 500 mg formulation of SQV was developed to meet the need for a more simplified ARV regimen with a reduced pill burden. The 500 mg tablet and 200 mg capsule offer equivalent bioavailability when administered at doses of 1000 mg in combination with 100 mg ritonavir (r) [7].

The efficacy and safety of SQV/r 1000/100 mg twice daily in combination with other ARVs have been established in randomized clinical trials [8–10]. However, while there are sufficient data on the treatment experience with the 200 mg SQV capsule [8,9], experience with the use of SQV 500 mg tablets at the FDA- and EMEA-approved dose is limited. The RAINBOW survey was initiated to establish a large database of patients being treated with the SQV 500 mg tablet at the approved dose of 1000 mg in combination with r 100 mg twice daily to evaluate the safety profile of SQV/r and to assess treatment response, as measured by CD4+ cell counts and HIV viral loads, in different patient subpopulations.

## Methods

### Study Design and Participants

This was an open-label, multicenter, phase 4 observational cohort study of HIV-infected patients 18 years of age or older who had been prescribed SQV/r 1000/100 mg twice daily with NRTIs or NNRTIs as part of their HIV treatment regimen. Excluded were patients who had received previous treatment with SQV, who had simultaneous treatment with a PI other than SQV/r, who had SQV/r at doses other than 1000/100 mg twice daily, or who had nonadherence or poor compliance to SQV/r that may have resulted in survey data being deemed unreliable.

All participants were prescribed SQV 1000 as 2 × 500 mg tablets twice daily. Survey data were transcribed by the investigator from the patient chart into an internet-based CRF system TRI@L-IT. In cases in which participants were being treated prior to enrollment, data were collected both retrospectively and prospectively. Baseline (before the first dose of SQV/r) demographic characteristics, hepatitis status, ARV history, laboratory data (including HIV RNA viral load, CD4+ count, and lipid levels), and clinical data were recorded as well as year of HIV diagnosis and current clinical disease stage and concomitant medications including lipid-lowering agents. The following data were collected from any visits occurring up to 24 weeks after initiation of therapy and were classified into the applicable time frame (weeks 2–8, weeks 8–16, and weeks 20–30): HIV RNA viral load, CD4+ cell count, dates of these measures, assays used and lower limit of detection, changes in concomitant medications including changes in or introduction to lipid lowering agents, unexpected and grade 3 and 4 adverse events (AEs) and relationship to the study specific medication, adherence information, and results of hematology and biochemistry tests. Safety analyses included the monitoring of changes in the following laboratory parameters from baseline to last available value: fasting lipids, bilirubin, glucose, alanine transaminase, aspartate transaminase, gamma-glutamyltransferase, and the percentage of patients receiving lipid-lowering agents.

This study was conducted in accordance with the Declaration of Helsinki in its revised edition (Edinburgh, Scotland, 2000) and the guidelines of current Good Clinical Practice. Local ethics committees approved the study protocol and documents relevant to their local regulatory requirements prior to recruitment to the survey. Written informed consent was obtained before any observational data were collected for inclusion in the survey. The right to request withdrawal of data from the study was reserved at any time.

### Analyses

The primary endpoint was the percentage of participants with a grade 3 or 4 AE. Secondary endpoints included the percentage of participants with <50 copies/mL and <400 copies/mL HIV RNA at week 24 and the increase in mean CD4+ cell count (cells/µL) from baseline to week 24. The efficacy analysis was based on the intent-to-treat population. Overall tolerability and efficacy (very good, good, sufficient, insufficient, cannot be evaluated) were assessed by the investigator at week 24. Analysis of safety and efficacy was also undertaken for the following subpopulations: treatment-naïve; treatment-experienced but naïve to PIs; switched from an alternative PI regimen (boosted lopinavir [LPV/r], atazanavir [ATV/r] or fosamprenavir [fAPV/r], or other PIs) due to toxicity; switched from a prior non-PI regimen due to toxicity; infected with hepatitis C virus (HCV); prior treatment at any time with a regimen that included a PI (LPV/r, AZV/r, f/APV/r or other); and prior treatment at any time with a regimen that did not include a PI. Participants could be included in more than one subpopulation. Note that these categories are not mutually exclusive, ie, a participant could have received a prior treatment regimen that included a PI as well as one that did not include a PI. In addition, AEs were analyzed according to gender, race/ethnicity, and country of residence. Adverse events, reported diseases, and medications were not coded but provided as listings of the original terminology. Participants who discontinued SQV/r treatment for any reason prior to week 24 were included in the survey as treatment failures.

A planned sample size of 2000 was selected as sufficient to gain representative information about treatment experience. Descriptive summary statistics were used in the analysis of this survey.

## Results

### Participant Disposition and Baseline Characteristics

**Figure 1:**
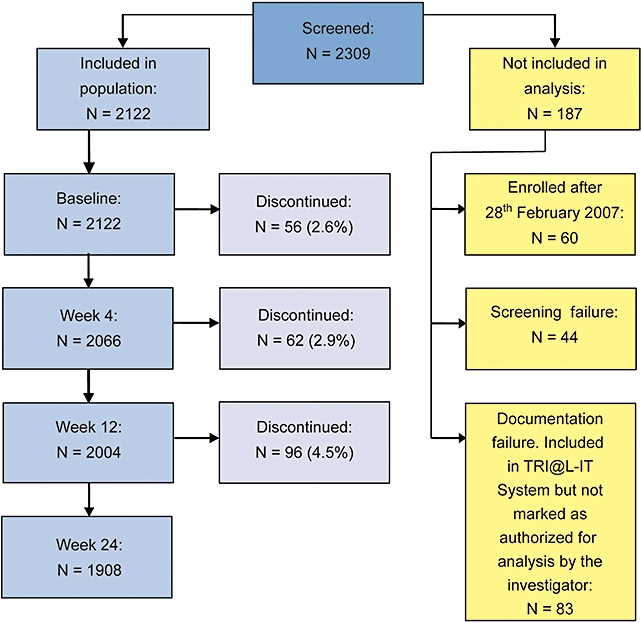
Participant disposition (“all participants” population). Since the recruitment phase of the survey was extended in Germany and Poland, participants from these countries who were enrolled after February 28, 2007, were not included in the global analysis. In addition, no overall evaluation of tolerability was available for those from centers in Germany since they were to be treated for longer than 24 weeks.

A total of 119 investigators at centers in Austria, Germany, Latvia, the Netherlands, Poland, Spain, Switzerland, and the United Kingdom enrolled 2309 participants between May 3, 2005 and September 20, 2007. Of those enrolled, 2122 were eligible for analysis and were included in the “all participants” population (Figure 1). The 187 participants not included in the analysis comprised: (i) 11 German participants and 49 Polish participants, countries where the recruitment date was extended beyond the original date of February 28, 2007; all participants recruited later than February 28, 2007 were excluded from the global analysis but included in the local trial analyses; (ii) 21 Spanish participants and 23 participants from the United Kingdom who were screened but ineligible; and (iii) 80 German participants, one Hispanic participant, and two Polish participants were included in the TRI@L-IT System but were either not marked as authorized for analysis by the investigator or excluded from analysis due to other documentation failure. Patients not marked as authorized for analysis were those for which the investigator did not complete the online data entry confirmation.

The number of participants in each subpopulation is presented in Table 1. In total, 78% were men with a majority (86%) being Caucasian; the mean age was 43 years (Table 2). The median baseline plasma HIV RNA viral load was 700 copies/mL, and the median CD4+ cell count was 323 cells/µL. The mean time since HIV diagnosis was 9 years (range: 0–35 years). In the “all participants” population, 31% had <50 copies/mL HIV RNA at baseline compared to only 1.5% of the treatment-naïve participants. Of the 2122 participants initially enrolled, 1908 completed the study. Reasons for premature withdrawal by subpopulation are shown in Table 3. While the percentages of participants withdrawn due to AEs (including nocturia, diarrhea, opportunistic infections, gastrointestinal intolerability, myocardial infarction, death, pneumococcemia, pregnancy, exanthema, vertigo, hyperglycemia, cachexia, erectile dysfunction, progression of lymphoma) were similar across treatment groups, there was a disproportionate percentage of participants lost to follow-up in the treatment-naïve and HCV coinfection subgroups; the reasons for this are not known.

**Table 1:** Subpopulations of participants initiating and completing the study, N (%)

	Participants initiating study*	Participants completing study
All participants	2122 (100.0)	1908 (89.9)
Subpopulation		
Treatment-naïve	413 (19.5)	360 (87.2)
PI-naïve but treatment-experienced	265 (12.5)	241 (90.9)
Toxicity switch from other PI regimen	161 (7.6)	151 (93.8)
Toxicity switch from prior non-PI regimen	201 (9.5)	183 (91.0)
HCV coinfection	287 (13.5)	254 (88.5)
Prior PI-containing regimen†	1386 (65.3)	1252 (90.3)
Prior non-PI-containing regimen†	1640 (77.3)	1485 (90.6)

* The percentage indicates the number of participants assigned to a particular subpopulation divided by the total study population. Since a participant may be counted in more than one subpopulation, the total percentage of the subpopulations exceeds 100%.

† At any time during disease therapy.

HCV = hepatitis C virus; PI = protease inhibitor.

**Table 2:** Demographic and baseline characteristics by analysis population

	All Participants (N = 2122)	Treatment-Naïve (N = 413)	PI-Naïve but Treatment- Experienced (N = 265)	Toxicity Switch from Other PI Regimen (N = 161)	Toxicity Switch from Prior Non-PI Regimen (N = 201)	HCV Coinfection (N = 287)	Prior PI (N = 1386)	Prior Non-PI Regimen (N = 1640)
Men, n (%)	1,662 (78.3)	313 (75.8)	196 (74.0)	128 (79.5)	152 (75.6)	201 (70.0)	1,120 (80.8)	1,308 (79.8)
Caucasian, n (%)	1,815 (85.5)	342 (82.8)	223 (84.2)	139 (86.3)	170 (84.6)	266 (92.7)	1,217 (87.8)	1,430 (87.2)
Age, mean years (range)	42.65 (18–83)	38.55 (18–69)	42.01 (21–79)	43.13 (18–68)	43.02 (23–75)	37.39 (18–58)	43.96 (18–83)	43.69 (18–83)
Weight, mean kg (range)	71.80 (1.58–148)	70.95 (35–110)	71.25 (1.58–109)	71.71 (43.5–98.3)	70.92 (43.5–100)	67.12 (41–125)	72.18 (42–148)	72.05 (1.58–148)
Duration of HIV infection, mean years (range)	8.83 (0–35)	3.12 (0–33)	8.37 (0–22)	9.76 (0–21)	9.97 (0–22)	9.46 (0–33)	10.63 (0–35)	10.30 (0–35)
HIV-1 RNA, copies/mL, median (25^th^– 75^th^ quartile)*	700 (49–57,834)	109,000 (30,700–370,000)	3,960 (49–47,458)	49 (49–399)	69 (49–18,450)	2,405 (49–66,300)	65 (49–6,330)	115 (49–12,400)
HIV-1 RNA <50 copies/mL, n (%)	662 (31.2)	6 (1.5)	63 (23.8)	89 (55.3)	87 (43.3)	69 (24.0)	585 (42.2)	645 (39.3)
HIV-1 RNA <400 copies/mL, n (%)	902 (42.5)	14 (3.4)	78 (29.4)	118 (73.3)	110 (54.7)	106 (36.9)	802 (57.9)	876 (53.4)
CD4+ cell count,* cells/µL, median (25^th^–75^th^ quartile)	323 (179–517)	187 (70–265)	321 (183–515)	386 (216–560)	400 (242–568)	244 (86–419)	367 (223–567)	362 (219–561)

* N varies. HCV = hepatitis C virus; PI = protease inhibitor.

**Table 3:** Participants withdrawn prematurely from treatment, N (%)

	All Participants	Treatment- Naïve	PI-Naïve but Treatment- Experienced	Toxicity Switch from Other PI Regimen	Toxicity Switch from Prior Non-PI Regimen	HCV Coinfection	Prior PI	Prior Non-PI Regimen
Participants Initiating Study	(N = 2122)	(N = 413)	(N = 265)	(N = 161)	(N = 201)	(N = 287)	(N = 1386)	(N = 1640)
Reason for withdrawal								
Adverse event	44 (2.1)	9 (2.2)	7 (2.6)	3 (1.9)	5 (2.5)	5 (1.7)	27 (1.9)	34 (2.1)
Lost to follow-up	42 (2.0)	17 (4.1)	1 (0.4)	2 (1.2)	1 (0.5)	12 (4.2)	23 (1.7)	23 (1.4)
Lack of adherence	26 (1.2)	5 (1.2)	4 (1.5)	1 (0.6)	4 (2.0)	7 (2.4)	17 (1.2)	20 (1.2)
Virologic failure	18 (0.8)	1 (0.2)	—	1 (0.6)	—	—	17 (1.2)	16 (1.0)
Intercurrent illness	11 (0.5)	4 (1.0)	2 (0.8)	—	2 (1.0)	—	5 (0.4)	7 (0.4)
Death	7 (0.3)	4 (1.0)	—	—	—	3 (1.0)	3 (0.2)	3 (0.2)
Other	43 (2.0)	9 (2.2)	5 (1.9)	1 (0.6)	6 (3.0)	3 (1.0)	28 (2.0)	33 (2.0)
None provided	23 (1.1)	4 (1.0)	5 (1.9)	2 (1.2)	—	3 (1.0)	14 (1.0)	19 (1.2)
All	214 (10.1)	53 (12.8)	24 (9.11)	10 (6.2)	18 (9.0)	33 (11.5)	134 (9.7)	155 (9.5)

HCV = hepatitis C virus; PI = protease inhibitor.

### Safety and Tolerability

There were 33 reports of grade 3 or 4 AEs in 29 (1.4%) participants in the “all participants” population (Table 4). Of these, 7 AEs reported in 7 participants (0.3%) were considered drug-related. The percentages of participants with grade 3 or 4 AEs were similar across subpopulations. The percentage of participants with all AEs also was similar across subpopulations (Table 5). There were 6 serious AEs resulting in 5 deaths (0.2%), all considered to be unrelated to treatment (3 due to lymphoma, 1 due to toxoplasmosis/miliar tuberculosis, and 1 due to suicide).

**Table 4:** Participants with grade 3 or 4 adverse events by analysis population (primary endpoint)

Type of Adverse Event	All Participants (N = 2122)	Treatment- Naïve (N = 413)	PI-Naïve but Treatment- Experienced (N = 265)	Toxicity Switch from Other PI Regimen (N = 161)	Toxicity Switch from Prior Non-PI Regimen (N = 201)	HCV Coinfection (N = 287)	Prior PI (N = 1386)	Prior Non-PI Regimen (N = 1640)
Grade 3/4, n	33	10	4	2	2	—	19	23
Participants, n (%)	29 (1.4)	7 (1.7)	4 (1.5)	2 (1.2)	2 (1.0)	5 (1.7)	18 (1.3)	22 (1.3)
Grade 3/4 treatment-related, n	7	—	—	—	2	—	7	7
Participants, n (%)	7 (0.3)	—	—	—	2 (1.0)	—	7 (0.5)	7 (0.4)

HCV = hepatitis C virus; PI = protease inhibitor.

**Table 5:** Summary of adverse events by analysis population

Type of Adverse Event	All Participants (N = 2122)	Treatment- Naïve (N = 413)	PI-Naïve but Treatment- Experienced (N = 265)	Toxicity Switch from Other PI Regimen (N = 161)	Toxicity Switch from Prior Non-PI Regimen (N = 201)	HCV Coinfection (N = 287)	Prior PI Regimen (N = 1386)	Prior Non-PI Regimen (N = 1640)
All adverse events	212	55	22	9	24	23	134	156
Participants, n (%)	141 (6.6)	34 (8.2)	15 (5.7)	9 (5.6)	17 (8.5)	16 (5.6)	91 (6.6)	106 (6.5)
All leading to death	6	4	—	1	—	2	2	2
Participants, n (%)	5 (0.2)	3 (0.7)	—	1 (0.6)	—	2 (0.7)	2 (0.1)	2 (0.1)
During treatment or within 28 days	197	52	21	9	23	21	123	144
Participants, n (%)	139 (6.6)	34 (8.2)	15 (5.7)	9 (5.6)	17 (8.5)	16 (5.6)	89 (6.4)	104 (6.3)
During treatment or within 28 days leading to death	6	4	—	1	—	2	2	2
Participants, n (%)	5 (0.2)	3 (0.7)	—	1 (0.6)	—	2 (0.7)	2 (0.1)	2 (0.1)
Treatment-related	100	24	9	3	12	6	67	76
Participants, n (%)	71 (3.4)	18 (4.4)	6 (2.3)	3 (1.9)	10 (5.0)	6 (2.1)	47 (3.4)	53 (3.2)
Serious adverse events	9	6	1	1	—	2	2	3
Participants, n (%)	8 (0.4)	5 (1.2)	1 (0.4)	1 (0.6)	—	2 (0.7)	2 (0.1)	3 (0.2)

HCV = hepatitis C virus; PI = protease inhibitor.

The incidence of AEs was comparable between men and women (6.7% vs. 7.4%) and across ethnicities. The highest occurrence of AEs by country was reported for participants from Spain while the lowest incidence (0.8%) was reported in those from Poland. The investigators rated the tolerability of SQV 500 mg as “very good” in 42% of participants, “good” in 25%, “sufficient” in 4%, and “insufficient” in less than 2% (Table 6). No information about the evaluation of tolerability was available for 463 (22%) participants because they continued to be followed in the subcohorts and subjective analysis was not carried out until the subjects left the survey.

**Table 6:** Overall evaluation of the tolerability of SQV 500 mg by analysis population, N (%)

	All Participants (N = 2122)	Treatment- Naïve (N = 413)	PI-Naïve but Treatment- Experienced (N = 265)	Toxicity Switch from Other PI Regimen (N = 161)	Toxicity Switch from Prior Non-PI Regimen (N = 201)	HCV Coinfection (N = 287)	Prior PI Regimen (N = 1386)	Prior Non-PI Regimen (N = 1640)
Very good	889 (41.9)	162 (39.2)	102 (38.5)	83 (51.6)	87 (43.3)	196 (68.3)	617 (44.5)	715 (43.6)
Good	535 (25.2)	88 (21.3)	68 (25.7)	32 (19.9)	47 (23.4)	43 (15.0)	375 (27.1)	441 (26.9)
Sufficient	90 (4.2)	14 (3.4)	8 (3.0)	9 (5.6)	6 (3.0)	5 (1.7)	66 (4.8)	74 (4.5)
Insufficient	35 (1.7)	6 (1.5)	4 (1.5)	4 (2.5)	5 (2.5)	7 (2.4)	25 (1.8)	29 (1.8)
Cannot be evaluated	110 (5.2)	21 (5.1)	18 (6.8)	6 (3.7)	13 (6.5)	22 (7.7)	70 (5.1)	86 (5.2)
No information	463 (21.8)	122 (29.5)	65 (24.5)	27 (16.8)	43 (21.4)	14 (4.9)	233 (16.8)	295 (18.0)

HCV = hepatitis C virus; PI = protease inhibitor; SQV = saquinavir.

Mean increases from baseline to 24 weeks in fasting plasma lipids are shown in Table 7. Mean triglyceride levels increased from baseline in the treatment-naïve and PI-naïve, treatment-experienced groups but only in the PI-naïve, treatment-experienced patient group were levels elevated above the normal range. Mean total cholesterol levels were elevated at baseline overall and in those receiving a prior PI regimen as well as a prior non-PI regimen and decreased slightly during the study. The treatment-naïve, PI-naïve, treatment-experienced, and toxicity switch from prior non-PI regimen treatment groups all had small increases from baseline in total cholesterol levels to greater than acceptable levels. Mean baseline fasting transaminase levels were elevated for all subgroups, with most having reductions at week 24.

**Table 7:** Baseline and change from baseline in fasting plasma lipids at week 24

	Triglycerides (normal <2.3 mmol/L)				HDL (normal <1.5 mmol/L [male], <1.7 mmol/L [female])				LDL (normal <4.2 mmol/L)				Total Cholesterol (normal <5 mmol/L)			
	Baseline		Change from Baseline		Baseline		Change from Baseline		Baseline		Change from Baseline		Baseline		Change from Baseline	
	Mean	N	Mean	N	Mean	N	Mean	N	Mean	N	Mean	N	Mean	N	Mean	N
All participants	2.64	1122	−0.02	841	1.18	908	0.08	656	2.91	806	0.19	581	5.11	1145	−0.03	844
Treatment-naïve	1.74	182	0.40	141	1.08	156	0.23	116	2.66	142	0.40	107	4.21	191	0.96	143
PI-naïve, but treatment-experienced	2.02	130	0.62	92	1.22	109	0.07	79	2.70	95	0.26	66	4.53	131	0.53	93
Toxicity switch from other PI regimen	2.66	101	−0.26	76	1.28	88	0.02	65	3.05	70	0.05	54	5.07	101	−0.11	76
Toxicity switch from prior non-PI regimen	2.63	112	0.13	86	1.21	97	0.02	78	2.96	86	0.41	66	4.97	114	0.26	88
HCV coinfection	1.90	120	−0.08	85	1.16	101	0.12	64	2.45	57	−0.13	36	4.21	145	0.11	97
Prior PI-containing regimen	2.95	809	−0.22	608	1.20	642	0.05	461	3.00	568	0.12	408	5.42	822	−0.35	608
Prior non-PI-containing regimen	2.80	933	−0.10	695	1.21	747	0.05	537	2.96	659	0.14	471	5.30	945	−0.25	696

HCV = hepatitis C virus; HDL = high-density lipoprotein; LDL = low-density lipoprotein; PI = protease inhibitor.

Mean fasting blood glucose levels were elevated at baseline in three treatment groups (normal range: 3.9–5.5 mmol/L): PI-naïve, treatment-experienced (7.63 mmol/L; N = 114), toxicity switch from prior non-PI regimen (8.04 mmol/L; N = 102), and HCV coinfected (7.31 mmol/L; N = 133). All had mean reductions resulting in normal levels by 24 weeks: PI-naïve, treatment-experienced (4.91 mmol/L; N = 93), toxicity switch from prior non-PI regimen (4.87 mmol/L; N = 88), and HCV co-infected (4.94 mmol/L; N = 109).

### Efficacy

**Figure 2:**
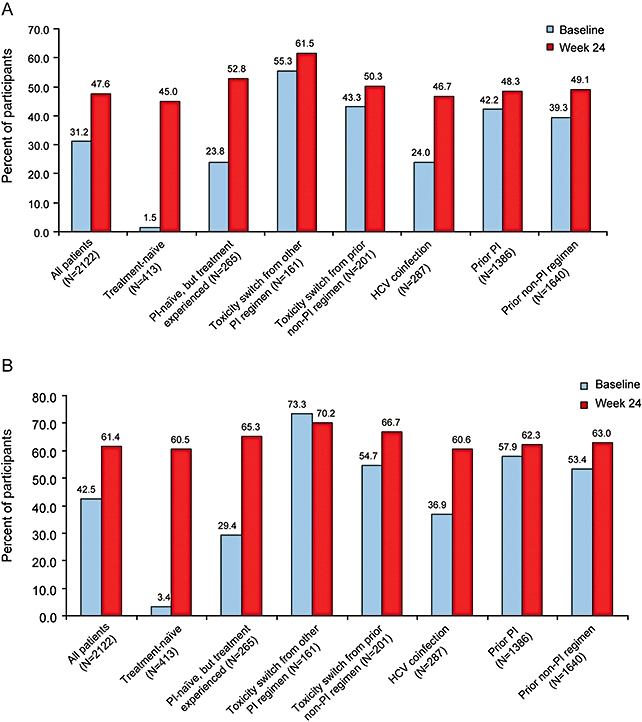
Percentage of participants in the intent-to-treat population with (A) HIV-1 RNA <50 copies/mL and (B) HIV-1 RNA <400 copies/mL at baseline and at week 24 overall and by subpopulation. HCV = hepatitis C virus; PI = protease inhibitor.

The percentages of participants in the intent-to-treat population with HIV RNA viral loads of <50 and <400 copies/mL at baseline and at week 24 for each of the subpopulations are shown in Figures 2A, B. In the overall population, 48% and 61% had <50 and <400 copies/mL at week 24, respectively. Participants who were treatment-naïve and PI-naïve achieved the greatest benefits with this regimen.

An increase in CD4+ cell count was also seen during the survey period in 523/734 (71%) of participants in the overall population having CD4+ cell count determinations (Table 8). The largest increase in CD4+ cell count (138 cells/µL) and the highest proportion of participants achieving an increase in CD4+ cell count (92%) occurred in the treatment-naïve subpopulation.

**Table 8:** Change in CD4+ cell count from baseline to week 24 and number (%) of participants with CD4+ cell count increases

	All Participants (N = 734)	Treatment-Naïve (N = 140)	PI-Naïve but Treatment- Experienced (N = 90)	Toxicity Switch from Other PI Regimen (N = 62)	Toxicity Switch from Prior Non-PI Regimen (N = 85)	HCV Coinfection (N = 57)	Prior PI Regimen (N = 503)	Prior Non-PI Regimen (N = 588)
Change in CD4+ cell count from baseline to week 24								
Median (25^th^–75^th^ quartile)	63	138	116	8	57	57	36	47
	(−19–169)	(63–236)	(22–258)	(−82–67)	(−38–207)	(3–148)	(−34–127)	(−32–139)
Number (%) of participants with increases in CD4+ cell count from baseline to week 24								
	523 (71.3)	129 (92.1)	71 (78.9)	34 (54.8)	57 (67.1)	44 (77.2)	322 (64.0)	390 (66.3)

HCV = hepatitis C virus; PI = protease inhibitor.

## Discussion

This observational survey demonstrated that treatment with the 500 mg film-coated tablet of SQV was well tolerated and effective in HIV-infected participants. Not only was there a very low incidence of grade 3 or 4 AEs, investigators rated the tolerability of the regimen as “very good” or “good” in 67% of their participants. A comparison of the efficacy across subpopulations showed that the majority had a favorable response to treatment, with those who were treatment- and PI-naïve deriving the most benefits. This is to be expected since these subpopulations would be most susceptible to a new regimen or to the addition of a new class of agent.

There were certain limitations to this survey, including its open-label design and its subjective assessment of tolerability (which could have biased the results) and the short duration of follow-up of 24 weeks. Still, the lack of blinding or of a control regimen was unlikely to bias the laboratory-based virologic efficacy endpoints. Furthermore, it was not possible to control for the variability in ARV regimens and populations across study sites. However, the value of this survey is its “real-world” clinical setting, which increases the level of heterogeneity of the enrolled participants and the concomitant ARV therapies. These results compare favorably with those of a recent study that showed that at 24 weeks, approximately 60% of trial nonparticipants (those in prospective, population-based cohort studies) achieved a viral load <500 copies/mL when initiating treatment with one of three PI-based regimens [11]. In contrast, at 24 weeks randomized controlled trial (RCT) participants had a much higher efficacy (approximately 75%). The authors suggested that reasons for this may include the strict inclusion criteria for RCT participants, the trial setting (better outcome in high-volume centers), and more frequent monitoring or stronger adherence incentives. In addition, a significantly higher percentage of RCT participants were ARV-naïve at baseline compared to trial nonparticipants (57% vs. 44%, respectively) [11].

The GEMINI study [10], an open-label, randomized, 48-week study, established the noninferior efficacy of using the SQV 500 mg tablet at approved doses (SQV/r 1000/100 mg twice daily) compared to LPV/r, both in combination with once-daily tenofovir/emtricitabine, in treatment-naïve HIV-infected participants. The proportions of patients with HIV RNA levels <50 copies/mL were 64.7% and 63.5% for the SQV/r and LPV/r treatment groups, respectively (estimated difference in proportion for noninferiority: 1.14%, 96% confidence interval: −9.6, 11.9; *P* < 0.012); triglyceride levels were significantly higher in the LPV/r group at week 48. The present study showed that in both treatment-naïve and treatment-experienced patients, oral SQV 500 mg was effective and well tolerated with a more convenient and practical dosing regimen than prior SQV-containing regimens.
